# Acute Exacerbation of Idiopathic Pulmonary Fibrosis With Pirfenidone and Nintedanib: A Friend or Foe

**DOI:** 10.7759/cureus.22606

**Published:** 2022-02-25

**Authors:** Mohit Kaushal, Dhruv Talwar, Deepak Prajapat, Sunil Kumar, Sourya Acharya, Deepak Talwar

**Affiliations:** 1 Department of Pulmonary and Critical Care Medicine, Metro Centre for Respiratory Disease, Noida, IND; 2 Department of Medicine, Jawaharlal Nehru Medical College, Datta Meghe Institute of Medical Sciences (Deemed to be University), Wardha, IND

**Keywords:** elderly patients, idiopathic pulmonary fibrosis, acute exacerbation, nintedanib, pirfenidone

## Abstract

Acute exacerbation (AE) in idiopathic pulmonary fibrosis (IPF) is unfortunate a deadly event with a very high mortality rate. Its occurrence is highly unpredictable, though few baseline risk factors have been identified. The revised definition of AE is more precise with clarity on defined parameters. However, no clear guidelines exist on treatment, with most therapies showing inconsistent benefits. Both the approved anti-fibrotic (pirfenidone and nintedanib) have shown equal efficacy in reducing the decline in lung functions, with few studies suggesting a drop in AE. We report a case of a patient with IPF with mildly impaired lung functions who was initiated on pirfenidone with dose titrated on a weekly basis but developed AE-IPF on day 10 of starting pirfenidone and after four days of doubling the dose from 600 mg/day to 1,200 mg/day. This raised the suspicion of whether pirfenidone played any role in this unfortunate event. With no response to conventional therapy of steroids and non-invasive ventilation for AE-IPF, initialization of nintedanib led to recovery with discharge of the patient in two weeks of hospitalization. This case highlights inadequacy in knowledge about the effects of these anti-fibrotics in IPF and recommends close monitoring in the future.

## Introduction

Acute exacerbation (AE) of idiopathic pulmonary fibrosis (IPF) is defined as a clinically significant deterioration in respiratory symptoms characterized by the presence of new widespread alveolar abnormalities [1]. The reported two-year incidence of AEs is 9.6%, with AE-IPF-related mortality being as high as 78% [2]. Baseline cardiovascular diseases, higher Gender-Age-Physiology (GAP) stage (≥II), and higher eosinophil percentage (≥3.21 %) in bronchoalveolar lavage (BAL) have been shown to be associated with the onset of AE of IPF [3,4]. Nintedanib has been shown to reduce the number of exacerbations in IPF [5]. AE-IPF after thoracic surgery for lung cancer has been reported, and pirfenidone has been used with some success preoperatively to prevent these exacerbations [6] and nintedanib has been used during treatment of AE-IPF in a case report [7]. However, both anti-fibrotics have not been shown to be associated with increased or inducing exacerbations. To the best of our knowledge, no case has been reported in which pirfenidone was implicated in inducing AE in IPF, though the possibility of chance occurrence of AE-IPF has been discussed.

## Case presentation

**Figure 1:**
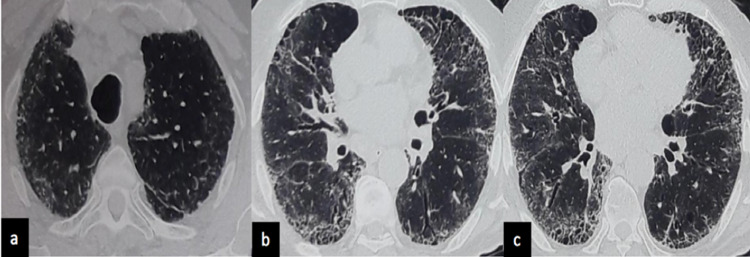
HRCT of the chest showing (a) inter-lobular, (b) intra-lobular, and (c) sub-pleural interstitial thickening involving the basal and peripheral regions, along with traction bronchiectasis and honeycombing. HRCT, high-resolution computed tomography

We present a case of an 84-year-old man, a reformed smoker from the last 12 years (bidi smoking index of 2000) with a history of hypertension and type 2 diabetes mellitus on regular treatment since the last 20 years. He presented to us with complaints of progressive breathlessness for the last three months (Modified Medical Research Council scale [mMRC] I-II), cough, and scant expectoration associated with a weight loss of around 6 kg. The patient was seen by his physician who noticed basal crackles and obtained a chest X-ray, which was reported as increased markings with bibasilar linear opacities, shaggy cardiac borders, and both diaphragms borders. The patient was further seen in our Interstitial Lung Disease Clinic and underwent high-resolution computed tomography (HRCT) of the chest with a complete evaluation of lung functions and autoimmune profile. There was no history of any known exposures. HRCT of the chest revealed inter-lobular, intra-lobular, and sub-pleural interstitial thickening involving the basal and peripheral regions, along with traction bronchiectasis, honeycombing, and mediastinal lymphadenopathy (largest node being 10 mm in short axis) (Figure 1) consistent with usual interstitial pneumonia (UIP) pattern.

Blood investigations revealed normal blood count (CBC), normal liver and renal function tests (LFT and KFT), and normal serum LDH (lactate dehydrogenase) levels (154U/L), with a negative autoimmune profile. His lung functions showed moderate restriction (forced vital capacity [FVC] 2.21L; 78% predicted) and significant de-saturation (21%) on the six-minute walk test (6MWT) with a distance covered of 224 meters (58% predicted).

**Figure 2:**
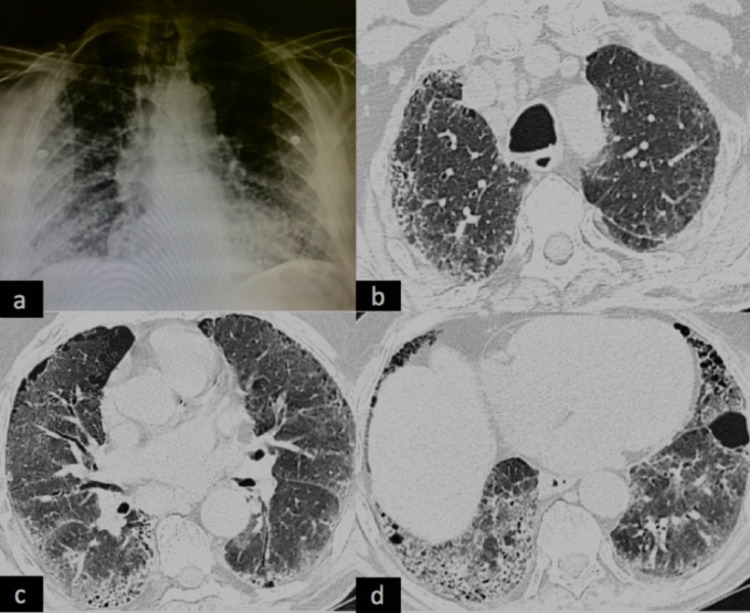
(a) Chest X-ray showing bilateral middle and lower zone noninhomogeneous opacities. (b-d) HRCT of the chest showing ground-glass opacities superimposed on UIP pattern. HRCT, high-resolution computed tomography; UIP, usual interstitial pneumonia

After multi-disciplinary discussion (MDD), a confident diagnosis of IPF (GAP I) was made. Although he had mild functional impairment, but in view of symptoms and to reduce AE, he was started on pirfenidone at 200 mg thrice a day, increasing to 400 mg thrice a day from day 7, and was advised review at two weeks with LFTs for further escalating the dose. He received standard supportive care with influenza and pneumococcal vaccinations and proton pump inhibitors. However, on day 10 of starting pirfenidone, he presented to the emergency department with an increase in breathlessness (mMRC grade IV) and baseline SpO_2_ of 85% at room air. There was no history of fever, and chest X-ray showed worsening with bilateral middle and lower zone non-homogeneous opacities (Figure 2a). Routine labs showed normal CBC, LFT, and KFT. D-dimer was 123 ng/mL, and repeat HRCT of the chest showed ground-glass opacities (GGOs) superimposed on UIP pattern seen earlier (Figures 2b-2d).

Echocardiography revealed a left ventricular ejection fraction (LVEF) of 55%, no left ventricular (LV) regional motion abnormality, right ventricle (RV) normal in size with adequate systolic function, and mild concentric LV hypertrophy. Sputum examination showed no acid-fast bacilli (AFB) and no growth on pyogenic cultures. Multiplex PCR (polymerase chain reaction) of sputum for viral and other infections was also negative. Pro-calcitonin (<0.05 ng/mL) and NT-proBNP (N-terminal pro-brain natriuretic peptide) levels (387pg/mL) were normal. His Krebs von den Lungen (KL-6) level was 3,867U/mL (normal ≤ 500 U/mL, with levels > 1,300 U/mL being a sensitive predictor for the onset of AE-IPF) [8].

**Figure 3:**
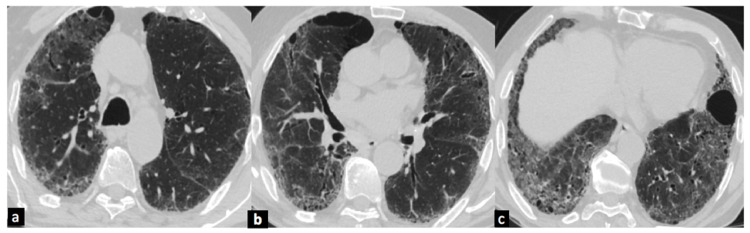
(a-c) HRCT chest showing marked improvement in ground-glass opacities in bilateral lung fields. HRCT, high-resolution computed tomography

The patient was diagnosed as having AE-IPF and was managed conservatively with parenteral methylprednisolone at 40 mg every six hours and parenteral amoxicillin-clavulanic acid, with non-invasive ventilation (NIV) support, oxygen, and other supportive treatment. However, the patient did not respond and continued to worsen. Family members declined consent for invasive mechanical ventilation (IMV). After discussion with family members, nintedanib 150 mg twice daily was added to his treatment regimen. The patient was gradually weaned from NIV over the next week and steroids were tapered off. The patient was discharged on day 16 on domiciliary oxygen and tapering steroids. The patient was followed in the outpatient department at two weeks, which showed marked improvement in GGOs on CT scan (Figure 3) and, then at six weeks, and reported improved symptoms with baseline SpO_2_ of 92% at room air.

## Discussion

AE-IPF is a major cause of morbidity and mortality in patients with IPF of unknown etiology and is reported as unpredictable acute worsening, which is not due to heart failure or fluid overload. It carries 40-60% hospital mortality with 90% mortality in six months following discharge [9,10]. Since infection has always been considered as one of the causes of pulmonary changes such as ARDS (acute respiratory distress syndrome), it needs to be ruled out prior to labeling as AE-IPF. Evidence has shown that clinical features and prognosis of AE-IPF are very similar to those of IPF exacerbation with a known cause such as pneumonia or aspiration [11]. Since exacerbation in both idiopathic and non-idiopathic disease results in the development of DAD (diffuse alveolar damage) on UIP pattern, the definition of AE-IPF has been revised, based on pathobiology, as the occurrence of acute lung injury with DAD regardless of the trigger condition [12,13].

Our case developed an AE of IPF within 10 days of initiating anti-fibrotic therapy with pirfenidone. AE in IPF is unpredictable, with a recent retrospective study reporting one- and three-year incidences rate of AE of 14.2% and 20.7%, respectively [9]. Hence, it is likely possibility that the occurrence of AE in our patient was a random event. The etiology of AE in IPF remains unknown with infections, micro-aspiration, drugs, massive transfusion, surgery, bronchoscopy, and BAL among a few likely triggers [14,15]. Even if idiopathic, the consequences and course of AE remain the same. The absence of all known triggers leads us to believe that the AE in our patient could be either idiopathic or pirfenidone-related as no other cause could be identified on detailed investigations. Drugs associated with the development of AE- IPF include biologicals (anakinra, etanercept, and infliximab) and nonbiologic (ambrisentan), as well as immune-modulatory (everolimus, interferon-alpha, and interferon-beta) and anti-neoplastic drugs (both conventional and targeted tyrosine kinase inhibitors). These variable triggers suggest different pathobiology for the occurrence of AE-IPF in various subsets of patients.

If we consider that AE-IPF, in this case, was idiopathic, we can look for baseline risk factors reported to predict future development of AE in IPF, such as lower FVC, never smoker status, and female sex. Another recent study showed baseline cardiovascular diseases, pulmonary hypertension, higher GAP stage (≥II), higher serum LDH level (≥180 U/L), and higher neutrophil (≥1.77 %) and eosinophil (≥3.21 %) percentages in BAL [3,4], or treatment with immune-suppressive before or after diagnosis being associated with fewer chances of AE-free course in IPF. None of these known factors were present in our case, thus decreasing the chances of this AE episode being idiopathic.

Since alveolar epithelial cell injury is the predominant factor in the pathogenesis of AE-IPF, we used the KL-6 marker, which is a sensitive biomarker for type II alveolar epithelial proliferation and/or injury [16] and predicts the short-term prognosis of rapid deterioration in IPF [17]. We did not have baseline KL-6 levels, but very high levels in our case confirm AE of recent origin.

Our case highlights AE of IPF while being initiated on pirfenidone and treated with nintedanib. AE-IPF episode is estimated to occur in 5%-10% of patients per year [18], with very high in-hospital mortality (up to 80%) in the past [10]. The use of NIV rather than IMV has resulted in a decline in mortality rates from 51.6% to 30.9%, and we also used NIV in our case [19].

Although it is debatable if AE in our patient occurred by chance when initiated on pirfenidone within 10 days, but as our patient had none of the known risk factors for AEs at baseline, possible implication of drug cannot be denied completely. Also, once AE-IPF sets in, there is no way to distinguish the cause behind it. We did not come across any such occurrence of AE-IPF with pirfenidone in safety studies in clinical trials as well as real-world experience. Also, prior use of pirfenidone has been shown to reduce postoperative AE in IPF cases undergoing thoracotomy for lung cancer [6]. But the time from the first visit to the onset of AE-IPF has been reported to be between 3 and 60 months [20] in the literature from various clinical trials, and the timeframe being only 10 days in our case supports an alternative view of pirfenidone being a likely player in an unknown way in triggering AE-IPF in this case. The case report of a patient on nintedanib who tolerated low dose but after increasing the dose to 150 mg twice a day and developed AE on day 65 [21] further supports our view that rapid up-titration of pirfenidone might have played a role in the development of AE in our case. Since in the aforementioned case report the patient had already failed on pirfenidone and it was not clear that nintedanib led to AE, authors could cautiously restart nintedanib. In our case, we did not have the option of reintroduction of pirfenidone as the patient did not improve and we tried the other anti-fibrotic nintedanib as few case reports of successful treatment of AE- IPF with nintedanib have been published in the literature [22].

Pirfenidone, a pyridone derivative, suppresses transforming growth factor (TGF)-beta induced myofibroblast differentiation and fibrogenic activity of human lung fibroblasts, though the exact mechanism of action in IPF is still unknown. The possible paradoxical effect of rapid dose escalation, thereby inducing worsening of our case, needs to be explored further. This would require in-depth analysis, as in the LOTUSS trial, a longer titration schedule (four weeks versus two weeks) was associated with better tolerability against a 14-day titration routinely recommended [23].

Clinical improvement in response to nintedanib being given during AE has been reported earlier also [22]. Our case was diagnosed as AE-IPF due to supportive clinical evidence including high levels of serum KL-6 along with characteristic changes witnessed on HRCT. As there was no cardiac or infective cause that could be identified and no response to conventional treatment, the patient was started on nintedanib. Good response in our case mirrors the reported benefits of its use in AE-IPF.

## Conclusions

The effect of pirfenidone in inducing AE-free period in IPF cases had conflicting results from various studies. The first trial was terminated early as there were AEs only in the placebo arm, but these results were not duplicated in the second trial. Safety data on anti-fibrotics are still being collected and submitted to regulators, and it would be worthwhile to relook from this perspective too as any patient of IPF developing AE would be considered as a failure of treatment rather than because of it. Hence, the impact of IPF therapies on risks and outcomes of AE in IPF needs further elucidation.
